# The influence and lag-effect of temperature and precipitation on the incidence and mortality of tuberculosis, 2000–2021: an observational study

**DOI:** 10.3389/fpubh.2025.1572422

**Published:** 2025-08-13

**Authors:** Qiao Liu, Yaping Wang, Min Liu, Yanlin Zhao, Jue Liu

**Affiliations:** ^1^Department of Epidemiology and Biostatistics, School of Public Health, Peking University, Beijing, China; ^2^Institute of Environmental Medicine, Peking University, Beijing, China; ^3^Key Laboratory of Epidemiology of Major Diseases (Peking University), Ministry of Education, Beijing, China; ^4^National Center for Tuberculosis Control and Prevention, Chinese Center for Disease Control and Prevention, Beijing, China; ^5^Institute for Global Health and Development, Peking University, Beijing, China

**Keywords:** temperature, precipitation, tuberculosis, lag effect, DLNM

## Abstract

**Background:**

Tuberculosis (TB) remains a major global health concern, particularly in low-and middle-income countries. Climate change may influence TB burden through effects on human health, living conditions, and pathogen transmission, yet its long-term impact remains underexplored.

**Methods:**

This observational study integrated data on temperature and precipitation obtained from NCEI/NOAA, TB burden from the Global Burden of Disease Study 2021, and socio-economic covariates from the World Bank open data platform. We used quasi-Poisson regression to assess non-lagged associations and applied distributed lag non-linear models to estimate lagged effects of climate exposure on age-standardized incidence and mortality rates (ASIR and ASMR) of TB from 2000 to 2021.

**Results:**

From 2000 to 2021, global TB age-standardized incidence rate and age-standardized mortality rate declined annually by 2.15 and 4.18%, respectively, with higher burdens in Africa and Southeast Asia. TB rates were elevated in males and those over 50, while younger age groups (<5, 5–14) in countries like the Philippines and Zimbabwe saw increases. A 1°C rise in temperature reduced age-standardized incidence rate by 0.89% and ASMR by 1.61%, while 1 mm increased precipitation raised age-standardized mortality rate by 1.80%, impacting males more. Higher temperatures increased TB rates in South-East Asia and Western Pacific, while precipitation raised rates in Africa, Eastern Mediterranean, and the Americas. Low and high temperatures showed negative lag effects after 12–15 years, while high temperatures posed a short-term risk for those aged 50+. 0 mm precipitation was protective after 10–15 years, while intermediate and humid precipitation levels had mixed effects, including some negative impacts on mortality.

**Conclusion:**

There is urgent need for tailored interventions that strengthen healthcare infrastructure, enhance disaster preparedness, and address both social determinants and climatic influences. By incorporating climate factors into the understanding of TB trends, our study offers critical insights to guide public health strategies in the era of climate change, contributing to more effective approaches for achieving the SDG targets for TB elimination.

## Introduction

The World Health Organization (WHO) underscored the persistent challenge of tuberculosis (TB) worldwide, with 10.8 million new cases and 1.25 million deaths in 2023 (1). The disease has likely surpassed COVID-19 as the leading cause of death from a single infectious agent globally. While there is a slight stabilization in the increase of new cases and a continued decline in deaths post-COVID-19, global targets for reducing TB incidence and mortality are far from being met (1). Regional disparities are stark, with the 30 high-burden countries accounting for 87% of all cases, significantly in South-East Asia (45%), Africa (24%), and the Western Pacific (17%) (1). Despite some progress in certain regions, the global milestones for reducing TB incidence and mortality are not on track, and the disease continues to exact a devastating toll, especially in areas with limited access to healthcare and robust TB control measures. Climate change further exacerbates the TB burden by influencing its patterns and dynamics through temperature, humidity, and precipitation changes. These factors alter host immunity and increase vulnerability, particularly among displaced populations (2). Extreme climatic events also disrupt TB diagnosis and treatment, facilitating disease transmission. However, evidence on this relationship remains limited, highlighting the need for further studies to better understand and address climate change’s impact on TB (2).

Temperature and humidity have been shown to influence TB incidence and mortality through complex mechanisms. A previous meta-analysis showed that TB risk was positively correlated with precipitation, while temperature, humidity, air pressure, and sunshine duration did not show statistically significant correlations (3). Low temperatures are frequently identified as risk factors for TB, with studies reporting increased TB incidence and hospitalizations during colder conditions, particularly in males and older populations (4–6). The effects of temperature often exhibit nonlinear and lagged patterns, with risks peaking months after exposure. For instance, temperatures between 16.3°C and 17.3°C were associated with increased TB notifications in Hong Kong, with risks peaking after 13–15 months (7). Conversely, high temperatures have demonstrated a negative association, reducing TB risk in certain settings (6, 8). Similarly, humidity influences TB risk through delayed effects, with moderate humidity levels associated with increased risks at specific lag times, such as 12–17 months after exposure in Hong Kong (7). However, short-term effects of humidity were often negligible or inconsistent (6). Importantly, the effects of temperature and humidity vary regionally and demographically. For instance, in Japan, temperature-related TB risks were heterogeneous across prefectures (4), while in China, older populations and those in polluted areas experienced greater risks (5, 6). Despite these findings, current research has notable limitations. Many studies focus on specific regions, limiting the generalizability of their results (4, 7, 8). The interactions between temperature, humidity, and other environmental or socioeconomic factors remain underexplored, and inconsistent methodologies hinder cross-study comparisons (5, 6, 9). Additionally, short study periods and inadequate consideration of demographic subgroups or climate change scenarios restrict the scope of current research (5, 6).

The adoption of the 17 Sustainable Development Goals (SDGs) in 2015 marked a significant milestone, with Goal 3 specifically targeting the promotion of healthy lives and well-being for all (10). Among its priorities is the aim to end epidemics of major diseases, including TB. In line with this, the WHO launched the End TB Strategy in 2014, aiming to reduce TB deaths and cases by 2030 and 2035 (11). Despite progress, challenges remain in achieving these ambitious targets, particularly as climate change continues to influence the spread and outcomes of infectious diseases like TB. This study seeks to explore the impact of temperature and precipitation on global TB incidence and mortality, analyzing variations across regions and age groups. By integrating climate factors into the understanding of TB trends, our research aims to provide valuable insights that will guide public health interventions, especially in the context of climate change, helping to shape more effective strategies to meet the SDG targets for TB elimination.

## Methods

### Study design and data source

This was an observational study which covered all the countries and territories that were TB-endemic and had reported daily temperature and precipitation from 2000 to 2021. We used meteorological data from the National Centers for Environmental Information (NCEI).^1^ NCEI is under the National Oceanic and Atmospheric Administration (NOAA) and provides environmental data, products, and services covering the depths of the ocean to the surface of the sun. We used TB data obtained from the 2021 Global Burden of Disease Study (GBD 2021) result tools.^2^ The GBD study consists of a systematic and scientific effort to quantify the comparative magnitude of health losses due to diseases by sex, age and location over time (12). We used data of socio-economic characteristics, socio-demographic characteristics, and health resources as covariates from the open database of the World Bank.^3^

### National annual average temperature and precipitation

We extracted the temperature and precipitation data from the NCEI website. The original data downloaded from the NCEI website were the daily temperature data and total precipitation data for a specific site on all dates within a year, recorded on a daily basis, but not all of them are 365 days, some are over 300 days, and some are only over a dozen days. We merged and processed the daily temperature and total precipitation data of all meteorological observation stations involved each year according to the year, and finally obtained the daily temperature and total precipitation data of global meteorological stations from 2000 to 2021.

### TB incidence and mortality data

Data was gathered from the GBD 2021 result tools, established by the GBD group (13). The general methodological approaches to estimate the incidence and mortality of TB were described elsewhere (14). Briefly, the estimation of TB incidence and mortality employs a multifaceted approach that integrates data from vital registration, surveillance, and verbal autopsies, adjusting for misclassifications and using statistical models like CODEm and DisMod-MR 2.1. This methodology accounts for risk-weighted latent TB infection prevalence, corrects for potential misclassifications, especially in children, and incorporates covariates such as TB strain prevalence and transmission risk. It also distinguishes between HIV-associated TB and drug-resistant forms of TB, applying spatiotemporal Gaussian process regressions and meta-regression models to predict proportions and generate consistent trends. Disability weights are assigned to reflect the severity of TB, and the entire process is calibrated against data from high-quality health systems to ensure accuracy (14).

Annual data from 2000 to 2021 of TB incidence rates and mortality rates with their 95% uncertainty interval (UI) were extracted. We reported TB incidence and death data in 204 countries and territories, which were classified into six WHO regions (African Region, Region of the Americas, Eastern Mediterranean Region, European Region, South-East Asia Region, and Western Pacific Region). Furthermore, we additionally reported the results by sex and five age groups, including <5 years, 5–14 years, 15–49 years, 50–69 years, and >70 years.

### Covariates

We used data of socio-economic characteristics, socio-demographic characteristics, and health resources as covariates. We extracted the Socio-demographic Index (SDI) from the website Global Health Data Exchange. The SDI is developed by GBD researchers and is a composite indicator of total fertility rate under age 25 years, years of education for those ages 15 and older, and lag distributed income per capita (15). Access to clean fuels and technologies for cooking (% of population) (ACT), Current health expenditure per capita (CHE), People practicing open defecation (% of population) (POD), People using at least basic drinking water services (% of population) (PBW), and People using at least basic sanitation services (% of population) (PBS) were all extracted from the World Bank.

### Statistical analysis

#### Estimated annual percentage change

The EAPC is a commonly used tool to quantified the rate trend over a specific interval (16, 17). A regression line was fitted to the natural logarithm of the rates (*y* = *α* + *βx*+*ε*, where y = ln (rate) and x = calendar year). EAPC was calculated as $\;(e^{\beta }-1)\times 100\%$, with 95% confidence intervals (CIs) obtained from the linear regression model. The term “increase” was used to describe trends when the EAPC and its lower boundary of 95% CI were both >0. In contrast, “decrease” was used when the EAPC and its upper boundary of 95% CI, were both <0. Otherwise, the term “stable” was used.

#### National annual average temperature and precipitation

For the temperature and precipitation data, based on the daily temperature and total precipitation values of all stations, we obtain the average of the daily temperature and total precipitation data for all days of each year, in order to obtain the annual average temperature and precipitation data for all stations. Finally, based on the geographic location of each meteorological observation station, we calculated annual average temperature and precipitation data of each country. We converted the units of temperature and precipitation into degrees Celsius and mm. Country–year units with missing temperature or precipitation records were excluded from the analysis. No imputation procedures were applied.

#### Quasi-Poisson generalized linear model

To explore the non-lag association between ASIR/ASMR and national annual average temperature and precipitation, we used the quasi-Poisson generalized linear model (quasi-Poisson GLM) (18) to calculate the effects. The choice of the quasi-Poisson model is driven by the nature of data, as ASIR/ASMR is a discrete variable, and it is often characterized by overdispersion. The formula was as follows:


$$\begin{array}{c} \log(Y(t))=\alpha +\beta_{1}AT_{\mathrm{ij}}+\beta_{2}AP_{\mathrm{ij}}+\beta_{3}\mathrm{SD}I_{\mathrm{ij}}+\beta_{4}\mathrm{AC}T_{\mathrm{ij}}+\beta_{5}\mathrm{CH}E_{\mathrm{ij}}\\ +\beta_{6}\mathrm{PO}D_{\mathrm{ij}}+\beta_{7}\mathrm{PB}S_{\mathrm{ij}}+\beta_{8}\mathrm{PB}W_{\mathrm{ij}}+\beta_{9}\text{Year} \end{array}$$

where Y(t) refers to the expected number of cases/deaths per 100,000 population on time t. Moreover, α refers to the intercept; β refers to the regression coefficient; AT refers to National annual average temperature (°C); AP refers to National annual average precipitation (mm); ACT refers to Access to clean fuels and technologies for cooking (% of population); CHE refers to Domestic general government health expenditure per capita (current US$); PBW refers to People using at least basic drinking water services (% of population); PBS refers to People using at least basic sanitation services (% of population); and year refers to calendar year. Effect size were indicated as a percentage change (%) in the number of cases/deaths per 100,000 population caused by each 1 unit change in AT/AP, calculated using the formula (e^β^-1)*100%.

To assess model adequacy, we evaluated overdispersion by examining the ratio of residual deviance to residual degrees of freedom (15.70) and the estimated dispersion parameter (20.72), both indicating substantial overdispersion and confirming the suitability of the quasi-Poisson specification. We further calculated the pseudo-R^2^ value (0.76), suggesting that the model explained a high proportion of the variance in tuberculosis outcomes. Multicollinearity among covariates was assessed using variance inflation factors, all of which were within acceptable limits (range: 1.07–5.21), indicating no serious multicollinearity (Supplementary Table 1).

#### Distributed lag non-linear model

Finally, we used the DLNM to estimate the lagged effects of AT and AP on TB incidence and mortality rates. This method allows for the modeling of both non-linear relationships and delayed effects over time, taking into account the potential for temperature and precipitation to have varying impacts across different time lags (19). By incorporating multiple lag periods, DLNM captures the dynamic nature of the association between environmental factors and TB outcomes, offering a more nuanced understanding of how temperature and precipitation influence disease incidence and mortality over time. The lag effects were also adjusted with SDI, ACT, CHE, POD, PBW and PBS.

## Results

### Global trends and distribution of TB incidence and deaths, 2000–2021

In 2021, the global age-standardized incidence rate (ASIR) and age-standardized mortality rate (ASMR) of TB were 103.00 (95% UI: 92.21–114.91) per 100,000 and 13.96 (95% UI: 12.61–15.72) per 100,000, respectively. Compared to 2000, these rates decreased annually by an average of 2.15% (95% CI: 2.04–2.25%) and 4.18% (95% CI: 4.11–4.26%), respectively. Somalia recorded the highest ASIR at 658.69 (95% UI: 568.35–753.72) per 100,000, followed by the Central African Republic at 595.99 (95% UI: 543.91–649.65) per 100,000 and Eritrea at 537.33 (95% UI: 470.66–608.26) per 100,000.

**Figure 1:**
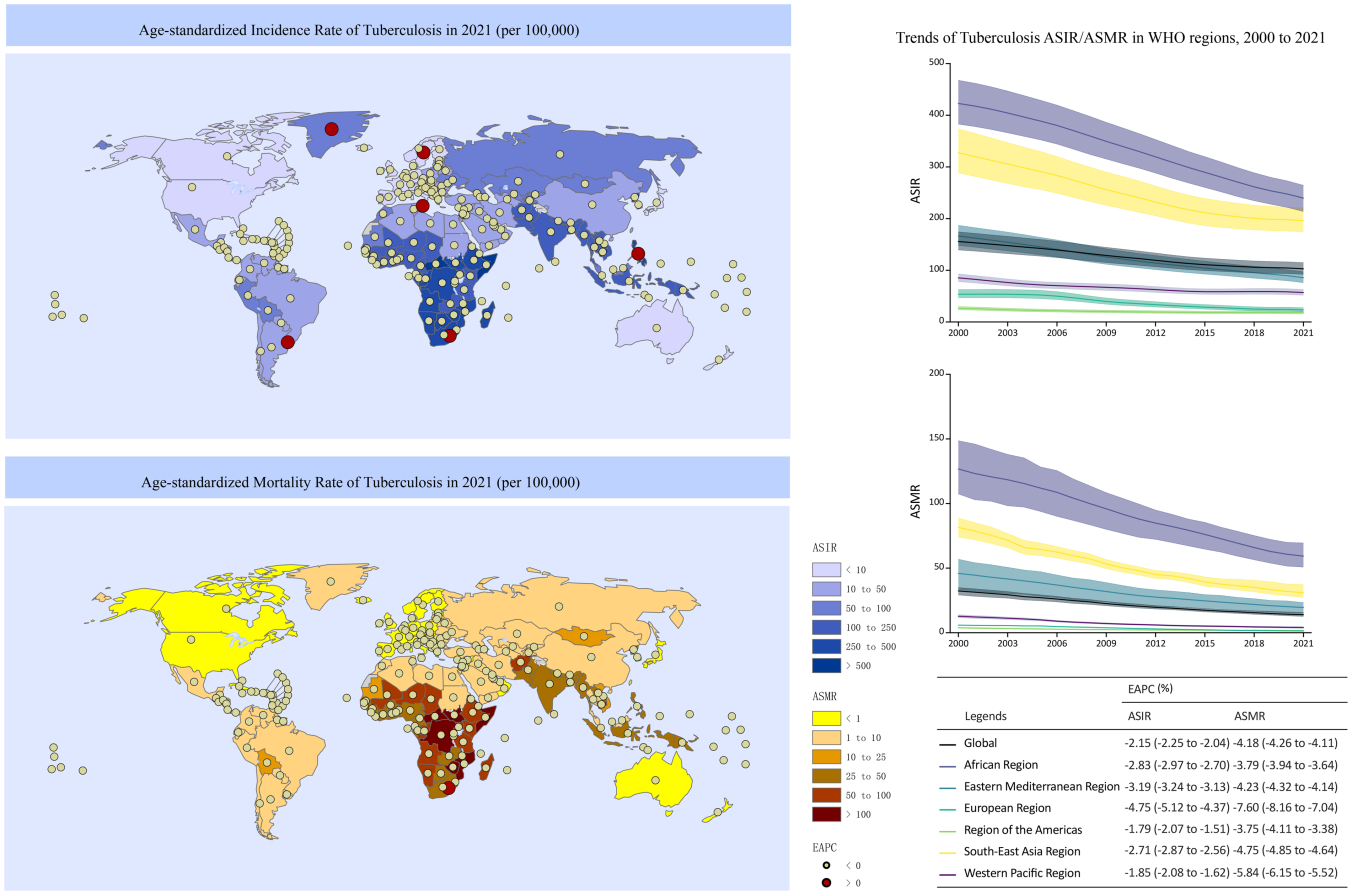
Tuberculosis ASIR/ASMR in 2021 and their trends between 2000 and 2021, nationally and regionally.

Most countries experienced a declining trend in TB ASIR between 2000 and 2021. However, five countries reported an increase, with the Philippines showing the fastest rise at an annual increase of 1.88% (95% CI: 1.52–2.23%), followed by Sweden (EAPC = 0.52, 95% CI: 0.29–0.74%) and Lesotho (EAPC = 0.46, 95% CI: 0.01–0.92%). The Central African Republic had the highest ASMR globally at 264.97 (95% UI: 173.66–356.79) per 100,000, closely followed by Somalia at 264.82 (95% UI: 158.28–456.76) per 100,000 and Lesotho at 213.42 (95% UI: 143.05–274.31) per 100,000. ASMR declined in all countries except Lesotho, where no significant change was observed (EAPC = 0.21, 95% CI: −0.60–1.03%) (Figure 1).

The ASIR and ASMR across all WHO regions showed a decreasing trend from 2000 to 2021. The European Region demonstrated the most rapid decline in ASIR and ASMR, with annual decreases of 4.75% (95% CI: 4.37–5.12%) and 7.60% (95% CI: 7.04–8.16%), respectively. The African Region and South-East Asia Region reported TB ASIR levels above the global average in 2021, at 239.76 (95% UI: 213.87–264.59) per 100,000 and 196.12 (95% UI: 174.57–220.58) per 100,000, respectively. The ASMR in the African Region, South-East Asia Region, and Eastern Mediterranean Region also exceeded the global average, at 59.31 (95% UI: 50.86–69.47), 31.01 (95% UI: 27.36–37.31), and 19.55 (95% UI: 15.14–23.35) per 100,000, respectively (Figure 1).

**Figure 2:**
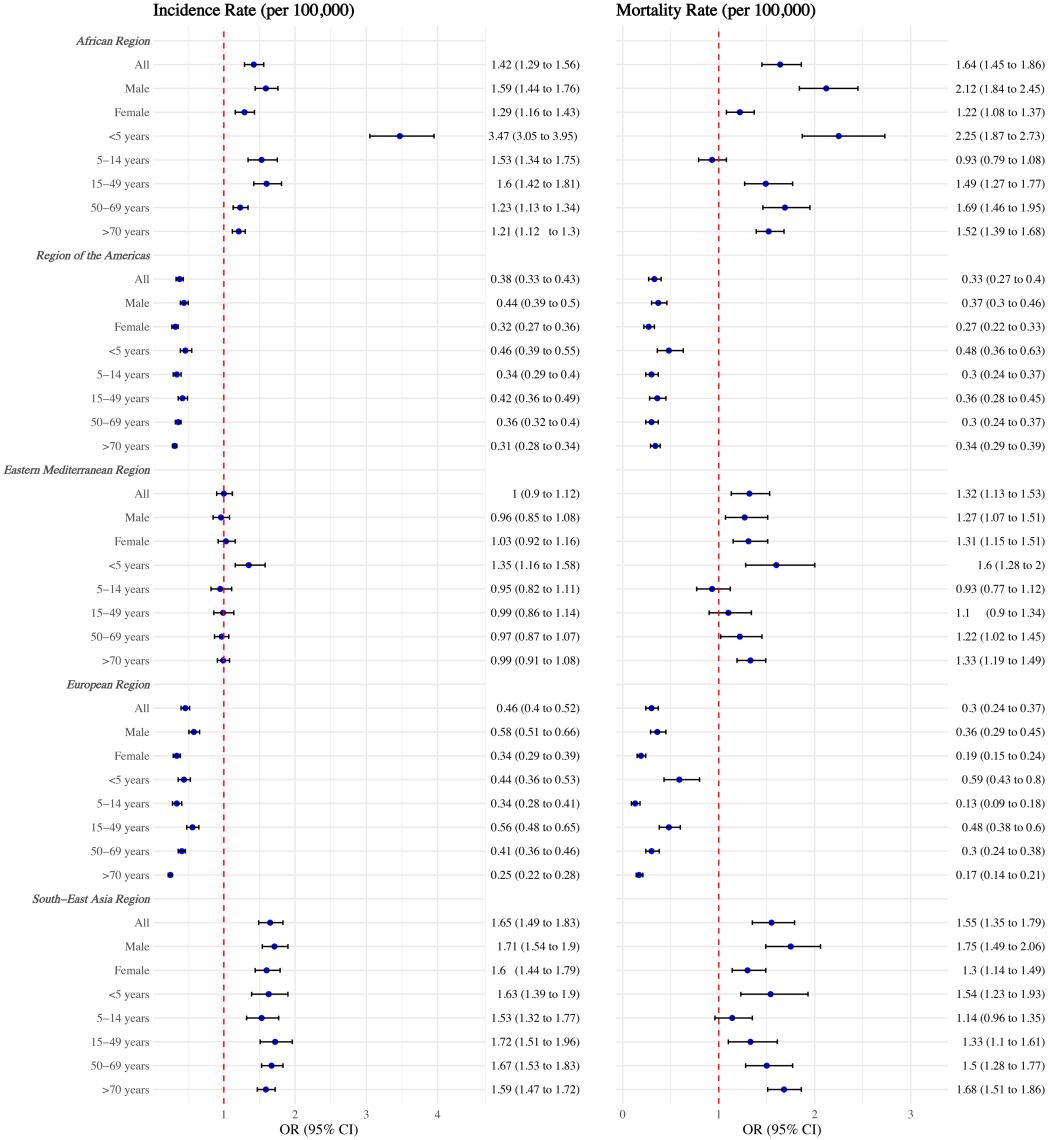
Tuberculosis incidence and mortality rates among WHO regions, overall, by sex, and by age group. Adjusted with National annual average temperature (°C), National annual average precipitation (mm), calendar year, Access to clean fuels and technologies for cooking (% of population), Domestic general government health expenditure per capita (current US$), People practicing open defecation (% of population), People using at least basic drinking water services (% of population), and People using at least basic sanitation services (% of population). No interaction terms were included.

As shown in Figure 2, significant regional disparities in TB incidence and mortality rates persisted after adjusting for various factors in the regression model. Children under five in the African Region had much higher TB incidence and mortality rates compared to other regions, with rates 3.47 times (95% CI: 3.05–3.95) and 2.25 times (95% CI: 1.87–2.73) higher than those in the Western Pacific Region, respectively. The TB burden in the South-East Asia Region remained the second highest globally.

### Age and sex distributions of TB incidence and deaths, by WHO region

**Figure 3:**
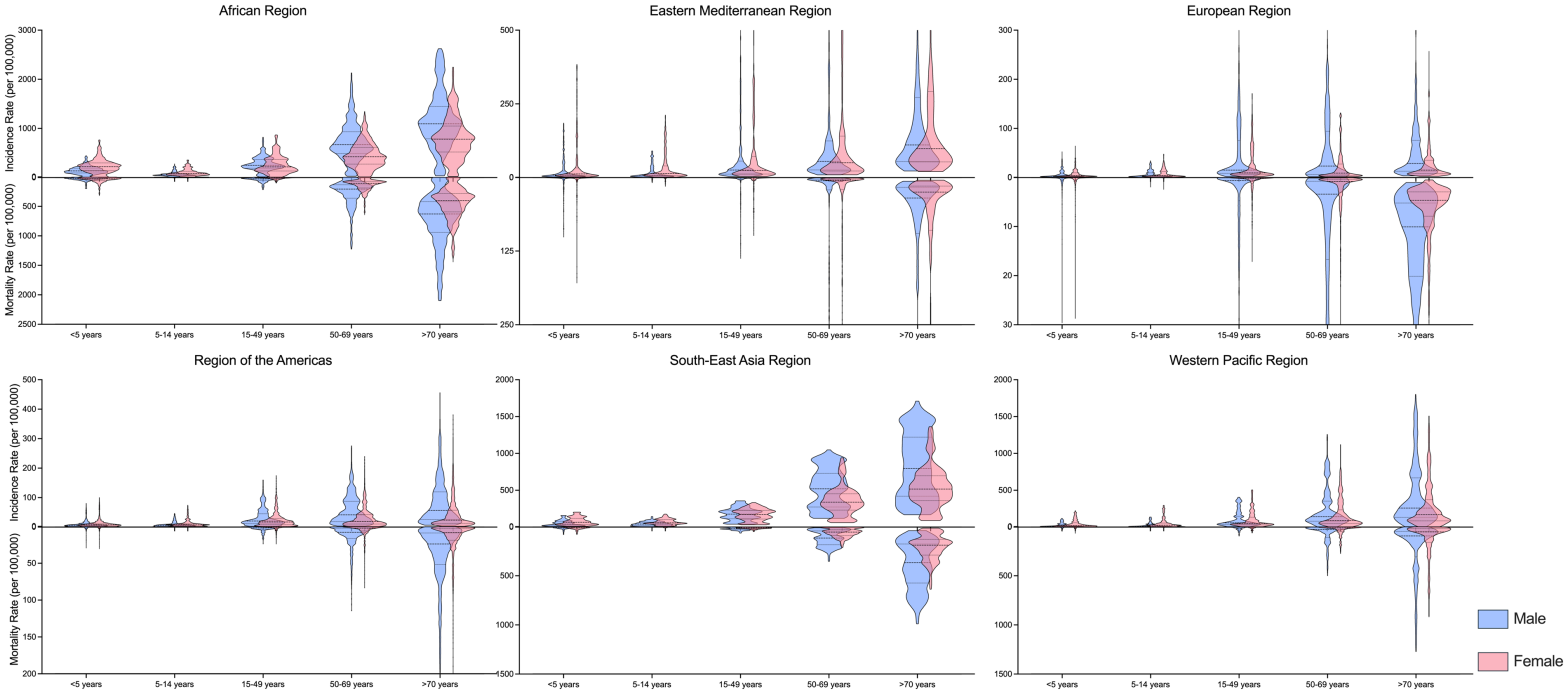
Age and sex disparities of tuberculosis incidence and mortality rate, in 2021, by WHO regions.

Both the incidence rate and mortality rate of TB were highest among individuals aged 50 years and older across all regions, with rates slightly higher among males compared to females (Figure 3). Although the incidence and mortality rates of TB declined across all sex and age groups within WHO regions from 2000 to 2021 (Supplementary Table 2), specific age groups in certain countries exhibited increasing trends in either incidence or mortality rates (Table 1).

**Table 1:** Countries and age groups with increasing trends of tuberculosis incidence/mortality rates between 2000 and 2021.

Location	Age group	EAPC (%) (95% CI)
Incidence rate		
Philippines	<5 years	9.64 (7.77–11.52)
Philippines	5–14 years	8.8 (7.22–10.39)
Philippines	70 + years	2.86 (1.95–3.77)
Philippines	15–49 years	1.41 (1.34–1.48)
Philippines	50–69 years	1.19 (0.77–1.61)
United States of America	<5 years	2.39 (0.41–4.36)
United States of America	5–14 years	1.01 (0.6–1.41)
South Africa	15–49 years	2.11 (1.74–2.48)
Uzbekistan	5–14 years	2.06 (0.6–3.53)
Uzbekistan	70 + years	0.6 (0.09–1.12)
Sweden	5–14 years	1.89 (1.52–2.27)
Sweden	15–49 years	1.48 (1.1–1.86)
Zimbabwe	<5 years	1.8 (1.26–2.34)
Lesotho	<5 years	1.59 (1.01–2.16)
Lesotho	15–49 years	0.99 (0.45–1.53)
Lesotho	5–14 years	0.33 (0.14–0.52)
Greenland	5–14 years	1.36 (0.32–2.4)
Malta	15–49 years	1.17 (0.75–1.59)
New Zealand	15–49 years	1 (0.41–1.6)
Uruguay	15–49 years	1 (0.69–1.3)
Eswatini	15–49 years	0.98 (0.64–1.33)
Eswatini	<5 years	0.72 (0.33–1.1)
Mozambique	15–49 years	0.96 (0.83–1.1)
Kyrgyzstan	70 + years	0.79 (0.44–1.14)
Central African Republic	15–49 years	0.6 (0.49–0.71)
Australia	15–49 years	0.59 (0.4–0.78)
Vanuatu	5–14 years	0.49 (0.31–0.67)
Canada	15–49 years	0.34 (0.05–0.62)
Guam	15–49 years	0.26 (0.17–0.35)
Canada	<5 years	0.24 (0.16–0.32)
Mortality rate		
Zimbabwe	5–14 years	4.42 (3.09–5.75)
Libya	<5 years	1.51 (0.72–2.3)
South Sudan	<5 years	1.06 (0.12–2)
Dominica	<5 years	0.74 (0.03–1.45)
Libya	15–49 years	0.42 (0.02–0.83)

In the Philippines, TB incidence rates increased across all age groups, with the most pronounced annual rises observed in the <5 years and 5–14 years age groups, at 9.64% (95% CI: 7.77–11.52%) and 8.80% (95% CI: 7.22–10.39%), respectively. In Zimbabwe, the TB mortality rate in the 5–14 years age group rose by an average of 4.42% annually (95% CI: 3.09–5.75%). Similarly, the mortality rate increased in the <5 years age group in Libya (EAPC = 1.51, 95% CI: 0.72–2.30%) and South Sudan (EAPC = 1.06, 95% CI: 0.12–2.00%) (Table 1).

### Global distribution of national annual average temperature, precipitation, and other covariates

From 2000 to 2021, the global national annual average temperature was 23.78°C (interquartile range: 13.72–27.28°C). The highest average temperature was observed in the Western Pacific Region at 27.23°C (25.65–28.08°C), while the European Region had the lowest, at 12.87°C (9.80–19.86°C). The global national annual average precipitation during the same period was 1.89 mm (interquartile range: 0.82–3.12 mm), with the Western Pacific Region recording the highest precipitation at 3.15 mm (interquartile range: 2.33–5.13 mm) and the Eastern Mediterranean Region the lowest at 0.50 mm (interquartile range: 0.14–2.18 mm). Additional covariates are detailed in Supplementary Table 3, revealing significant regional disparities in their distribution across WHO regions.

### The effect of annual average temperature and precipitation on TB

**Figure 4:**
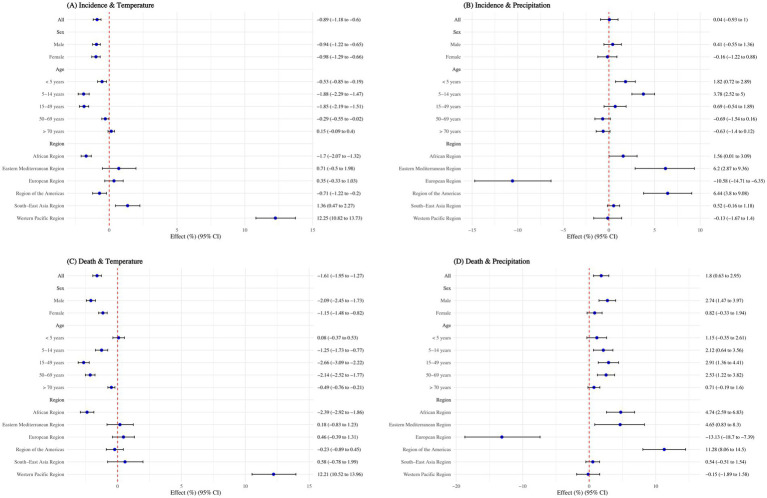
Effect of annual average temperature and precipitation on tuberculosis, overall and by sex, age group, and WHO regions. **(A)** Incidence and temperature; **(B)** Incidence and precipitation; **(C)** Death and temperature; **(D)** Death and precipitation. Adjusted with calendar year, Access to clean fuels and technologies for cooking (% of population), Domestic general government health expenditure per capita (current US$), People practicing open defecation (% of population), People using at least basic drinking water services (% of population), and People using at least basic sanitation services (% of population). No interaction terms were included.

Without considering lag effects, globally, each unit increase in national annual average temperature was associated with a 0.89% reduction in TB ASIR (95% CI: 0.60–1.18%) and a 1.61% reduction in ASMR (95% CI: 1.27–1.95%). However, each unit increase in national annual average precipitation had no significant effect on ASIR but resulted in a 1.80% increase in ASMR (95% CI: 0.63–2.95%). The effects of temperature and precipitation on TB incidence and mortality rates were similar across sexes, except for precipitation’s impact on ASMR: each unit increase in precipitation caused a 2.74% increase in male ASMR (95% CI: 1.47–3.97%), with no significant effect on females (Figure 4).

When stratified by age, the negative association of temperature on TB incidence rate was not significant for individuals aged >70 years (*p* > 0.05). For those aged 50–69 years, the negative association was minimal, with a 0.29% reduction (95% CI: 0.02–0.55%) in incidence rate per unit increase in temperature. Regarding mortality rates, the negative association was not significant for children aged <5 years (*p* > 0.05) and was smallest for those aged >70 years, with a 0.49% reduction (95% CI: 0.21–0.76%) per unit increase in temperature. Although precipitation had no significant overall impact on TB, each unit increase in precipitation led to a 1.82% increase (95% CI: 0.72–2.89%) in incidence rate for children aged <5 years and a 3.78% increase (95% CI: 2.52–5.00%) for those aged 5–14 years. It also caused mortality rates to rise by 2.12% (95% CI: 0.63–3.56%) for the 5–14 years age group, 2.91% (95% CI: 1.36–4.41%) for the 15–49 years age group, and 2.53% (95% CI: 1.22–3.82%) for the 50–69 years age group (Figure 4).

The relationship between temperature, precipitation, and TB varied significantly across WHO regions. Each degree increase in temperature has negative association for ASIR in the African Region and Region of the Americas, reducing it by 1.70% (95% CI: 1.32–2.07%) and 0.71% (95% CI: 0.20–1.22%), respectively. However, in the South-East Asia Region and Western Pacific Region, higher temperatures were risk factors for ASIR, increasing it by 1.36% (95% CI: 0.47–2.26%) and 12.25% (95% CI: 10.82–13.73%), respectively. For ASMR, a one-degree increase in temperature reduced rates by 2.39% (95% CI: 1.86–2.92%) in the African Region but increased rates by 12.21% (95% CI: 10.52–13.96%) in the Western Pacific Region.

Regarding precipitation, each 1 mm increase significantly reduced ASIR by 10.58% and ASMR by 13.13% in the European Region (*p* < 0.05). However, in the African Region, Eastern Mediterranean Region, and Region of the Americas, precipitation acted as a risk factor, increasing ASIR by 1.56–6.44% and ASMR by 4.65–11.28% (all *p* < 0.05) (Figure 4).

### The lag effect of annual average temperature and precipitation on TB

**Figure 5:**
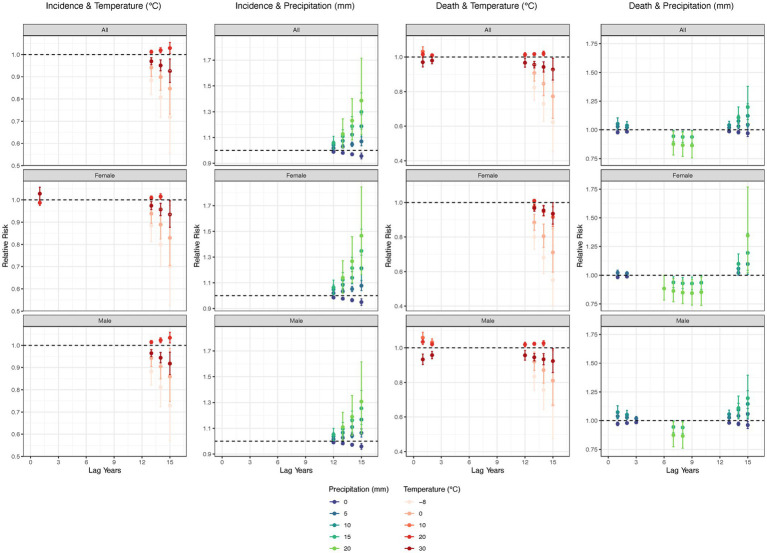
Lag effect of annual average temperature and precipitation on tuberculosis, overall and by sex. Adjusted with Access to clean fuels and technologies for cooking (% of population), Domestic general government health expenditure per capita (current US$), People practicing open defecation (% of population), People using at least basic drinking water services (% of population), and People using at least basic sanitation services (% of population). No interaction terms were included. Only statistically significant results were displayed.

Overall, the lag effects of low and high temperatures (−8, 0, and 30°C) on TB incidence and mortality rates showed a negative influence after 13 years. For intermediate temperatures (20°C), the lag effects showed a risk effect beyond 13 years. Drought conditions (0 mm precipitation) exhibited a protective factor for both incidence (after 12–15 years) and mortality rates (within 3 years and after 13 years). Humid conditions (20 mm precipitation) showed a risk lag effect on ASIR after 12 years, but demonstrated a negative association on ASMR after 6–10 years. Moderate levels of precipitation (5, 10, and 15 mm) had lag effects opposite to those of drought (Figure 5).

**Figure 6:**
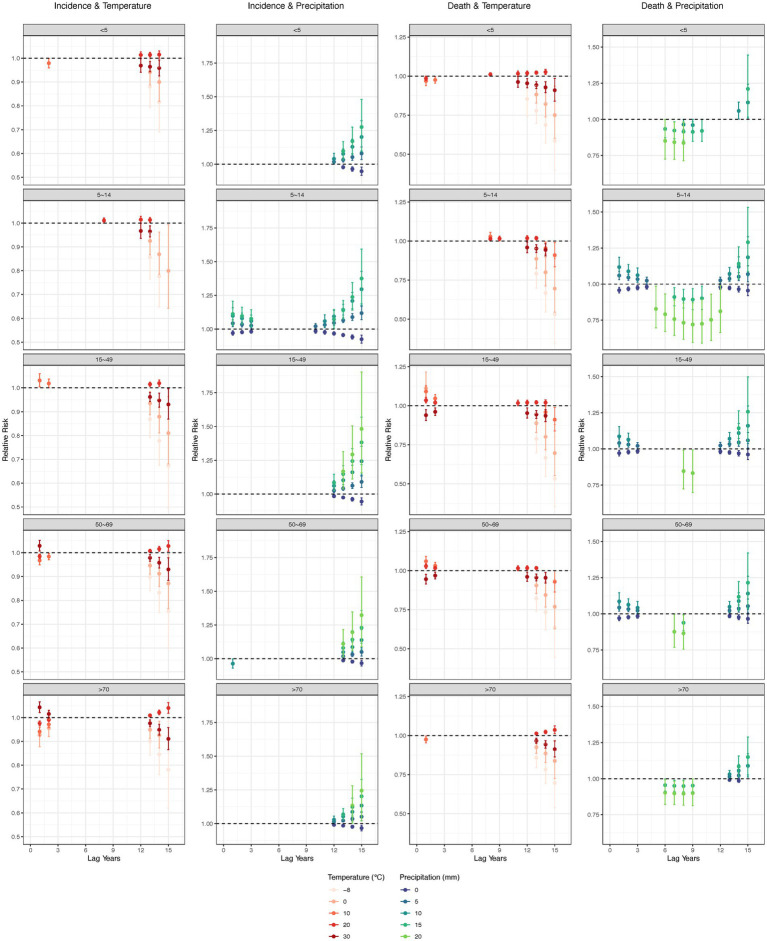
Lag effect of annual average temperature and precipitation on tuberculosis, by age groups. Adjusted with Access to clean fuels and technologies for cooking (% of population), Domestic general government health expenditure per capita (current US$), People practicing open defecation (% of population), People using at least basic drinking water services (% of population), and People using at least basic sanitation services (% of population). No interaction terms were included. Only statistically significant results were displayed.

For both low temperatures (−8 and 0°C) and high temperatures (30°C), the negative association on TB incidence and mortality was seen after 12 years, and peaked at 15 years. However, for people aged 50+, high temperature became a risk factor after the first year. Dry conditions (0 mm precipitation) had a negative lagged effect on TB incidence in all age groups after 10 years, while other precipitation levels posed a risk. The effect of precipitation on mortality was similar to that on incidence, however, 20 mm precipitation was negatively associated with all age groups between 5 and 10 years (Figure 6).

## Discussion

To the best of our knowledge, this was the first effort to comprehensively analyze global distribution of TB incidence and mortality from 2000 to 2021, and evaluate the influence, as well as the lagged effects, of temperature and precipitation on TB incidence and mortality globally, and across different regions and age groups. From 2000 to 2021, the global annual average declines in TB ASIR and ASMR were 2.15 and 4.18%, respectively. The African and Southeast Asian regions experienced higher burdens than others. TB incidence and mortality rates were notably higher in those over 50, especially in males. In countries like the Philippines, Zimbabwe, Libya, and South Sudan, certain age groups (<5 and 5–14 years) showed increasing TB rates. A 1°C increase in national annual average temperature was linked to a 0.89% reduction in ASIR and a 1.61% reduction in ASMR. 1 mm increased precipitation led to a 1.80% increase in ASMR, with males more affected than females. Temperature’s negative association was weaker for older age groups, while increased precipitation raised TB rates in younger groups (<5 and 5–14 years) and adults aged 15–69 years. In the South-East Asia and Western Pacific regions, higher temperatures increased TB incidence and mortality. In the African, Eastern Mediterranean, and Region of the Americas, increased precipitation was associated with higher TB incidence and mortality. Lag effect of low and high temperatures generally showed a negative association on TB incidence and mortality after 12–13 years, peaking at 15 years, though high temperatures became a risk for individuals aged 50 + after the first year. Drought conditions (0 mm precipitation) had a negative lag effect on incidence after 10–15 years, while intermediate and humid precipitation levels exhibited risk effects, with some negative associations on mortality observed at specific timeframes (e.g., 20 mm after 5–10 years). These findings underscore the complex and regionally variable interactions between climatic factors and TB dynamics, providing critical insights for tailoring public health strategies to mitigate the impacts of climate change on TB burden globally.

Our findings showed that from 2000 to 2021, global TB ASIR and ASMR declined annually by 2.15 and 4.18%, respectively, but exceptions included rising ASIR in the Philippines, persistent high burdens in Africa and Southeast Asia, and increasing tuberculosis rates in younger age groups (<5 and 5–14 years) in specific countries such as Zimbabwe and South Sudan. To address the global disparities in tuberculosis burden, targeted policy interventions are needed. High-burden regions, particularly in Africa and Southeast Asia, should receive increased financial and healthcare support to strengthen tuberculosis prevention, diagnosis, and treatment programs (20). Countries like the Philippines, where tuberculosis incidence is rising, require targeted assistance to improve early detection and healthcare access. Specific interventions should also focus on vulnerable age groups, such as children and adolescents in Zimbabwe and South Sudan, with enhanced vaccination and healthcare access (21). For low-burden countries, continued support in maintaining robust surveillance systems and preventative measures is crucial. Urgent action is needed to address the social and economic determinants of TB, such as poverty, malnutrition, HIV infection, smoking, and diabetes, while strengthening global collaboration, data monitoring, and knowledge-sharing to ensure effective interventions that reduce incidence and mortality and tackle the health inequities evident in the regional distribution of cases (1).

A 1°C increase in national annual average temperature was associated with a significant reduction in both TB ASIR by 0.89% and ASMR by 1.61%. The negative association of increased temperature on TB incidence and mortality rates was consist with previous studies (4–6, 8). However, the negative association was less pronounced for older age groups, a finding that highlights a growing concern for geriatric TB. With the global aging population, tuberculosis in older adults is becoming an increasingly important clinical and public health challenge, particularly in many Asian countries in the eastern and southeastern regions (22, 23). Understanding the mechanisms underlying TB in the older adults could lead to better prevention strategies, including interventions to prevent reactivation of TB, as well as improved treatment regimens and management approaches (24). Additionally, addressing the social determinants of geriatric TB—such as poverty, access to healthcare, and social isolation—is essential for improving patient outcomes and controlling the disease on a broader scale.

In the South-East Asia and Western Pacific regions, higher temperatures were found to be associated with increased tuberculosis (TB) incidence and mortality, with a 1°C rise in national annual average temperature corresponding to a 12.25 and 12.21% increase in TB ASIR and ASMR, respectively, particularly in the Western Pacific region. Despite the general negative associations of rising temperatures in other areas, the increase in TB in these regions can be attributed to several factors. These areas already bear a high TB burden, making the population more vulnerable to environmental changes. In the Western Pacific, population aging, persistent urban overcrowding, and climate-sensitive comorbidities—such as diabetes and chronic respiratory conditions—may amplify the health impacts of warming (25). Warmer temperatures may worsen health conditions such as malnutrition, HIV co-infection, and other comorbidities, increasing vulnerability to TB, while socio-economic factors like overcrowding, poor sanitation, and limited healthcare access exacerbate its spread (22, 26, 27). Moreover, public health infrastructure in several Western Pacific countries remains uneven, with gaps in active TB case detection, preventive therapy coverage, and social protection, which may hinder the adaptive capacity to rising climate risks (28). In regions with already warm climates, rising temperatures may enhance the survival and transmission of *Mycobacterium tuberculosis* in the environment and impair human metabolic functions, further elevating TB risks (2). Poor air quality and malnutrition, aggravated by warm and humid conditions, alongside under-resourced healthcare systems, compound these challenges, leading to higher TB incidence and mortality rates (29, 30). These region-specific vulnerabilities highlight the need to interpret climate–TB associations in the context of broader health system resilience, demographic patterns, and environmental stressors. Thus, strengthening healthcare infrastructure and addressing socio-economic determinants, such as improving housing and sanitation, are essential to mitigate the impact of climate change on TB in these vulnerable regions.

While increased precipitation did not significantly affect TB incidence, it was associated with a significant 1.80% increase in TB mortality. Male ASMR was more affected by precipitation than female ASMR. Increased precipitation led to higher TB incidence rates in younger age groups (<5 and 5–14 years) and mortality rates in median aged people (5–69 years). In African, Eastern Mediterranean, and Region of the Americas, increased precipitation was associated with higher TB incidence and mortality. This relationship could be attributed to the impact of humid environments and floods on the survival and transmission of *Mycobacterium tuberculosis*. Flooding can displace populations, create overcrowded shelters with poor ventilation, and disrupt access to healthcare, all of which facilitate TB transmission (31). Humid conditions can prolong the viability of *M. tuberculosis* in aerosol droplets, enhancing its spread in densely populated areas (32, 33). To address these challenges, it is essential to improve disaster preparedness and response, particularly in high-risk regions, by strengthening healthcare infrastructure, ensuring access to TB treatment, and promoting better housing and ventilation in flood-prone areas.

Lag effects of low and high temperatures generally showed a negative influence on TB incidence and mortality after 12–15 years, though high temperatures posed a risk for individuals aged 50 + within the first year. Drought conditions (0 mm precipitation) demonstrated a negative lag effect on TB incidence across all age groups after 10–15 years, possibly due to reduced exposure to damp environments that facilitate *M. tuberculosis* transmission (34). However, intermediate and humid precipitation levels were associated with risk effects, with some negative impacts on mortality observed at specific periods (e.g., 5–10 years for high precipitation). While TB pathogenesis typically involves a shorter latency period, these long-term effects may reflect broader structural and population-level mechanisms triggered by sustained climatic exposures, such as gradual changes in housing quality, nutrition, environmental degradation, and health system development (34). These findings highlight the importance of age-specific and climate-sensitive interventions, such as targeted vaccinations, nutritional support, and improved housing to mitigate TB risks. Integrating TB prevention strategies into climate-adaptive healthcare and disaster risk management plans remains essential for reducing TB’s long-term burden in vulnerable populations.

## Limitation

Our study had some limitations. First, this was an ecological study linking aggregated TB and meteorological data at the national level, which may introduce ecological fallacies—that is, associations observed at the population level may not hold true for individuals. This design limits causal inference and may obscure the nuanced pathways through which climatic factors influence TB risk. Second, the limitations of using the GBD database are unavoidable: only yearly and national-level data were available, and when original data were sparse or missing, the disease burden was estimated through modeling. Third, although we adjusted for several key socioeconomic and environmental indicators—including the SDI, health expenditure, and access to sanitation and clean water—some known determinants of TB risk were not included, such as air pollution (e.g., PM2.5), HIV prevalence, TB vaccine coverage, urbanization, and detailed healthcare access metrics. Notably, HIV prevalence may act as both a confounder and a mediator in the climate–TB pathway and is also strongly correlated with multiple covariates already included in our model (e.g., SDI, sanitation, and health system indicators), raising concerns of multicollinearity or over-adjustment. Additionally, global HIV and air pollution data are inconsistently reported across countries and years. Therefore, we did not include HIV prevalence or other such variables to avoid biased or unstable estimates. Fourth, the study’s reliance on national-level data may mask significant subnational heterogeneity in both climatic exposures and TB burden, which may differ greatly within countries by region, socioeconomic status, and health system capacity. Future research should consider integrating subnational or individual-level data, where available, to more accurately capture spatial and demographic variations and strengthen causal interpretation. Despite these limitations, our findings provide valuable insights into the complex interplay between climate change and TB burden, highlighting the need for climate-adaptive health strategies and targeted interventions to mitigate future risks.

## Conclusion

This study underscores the significant and regionally variable influence of climatic factors on TB trends. While global TB ASIR and ASMR declined overtime, persistent burdens remain in high-risk regions and vulnerable age groups. Rising temperatures were generally associated with decreased TB incidence and mortality, yet exhibited opposite effects in South-East Asia and Western Pacific, where warming correlated with increased TB burden. Increased precipitation was linked to higher TB mortality, particularly in males and younger populations. Lagged climatic effects revealed age-specific vulnerabilities, with short-term heat risks in older adults and longer-term impacts of drought (0 mm precipitation) showing negative associations after 10–15 years.

These results highlight the complex interplay between climate and TB dynamics and reinforce the urgency of implementing region-specific, climate-adaptive public health strategies. Strengthening healthcare infrastructure, improving early warning systems, and addressing social determinants in the context of environmental change are essential. By integrating climate considerations into TB control planning, our findings provided valuable insights for advancing global TB elimination goals and aligning efforts with the SDGs.

## Data Availability

The original contributions presented in the study are included in the article/Supplementary material, further inquiries can be directed to the corresponding author.

## Footnotes

^1^ https://www.ncei.noaa.gov

^2^ http://ghdx.healthdata.org/gbd-results-tool

^3^ https://databank.worldbank.org/
