# Supplementary material for: The influence and lag-effect of temperature and precipitation on the incidence and mortality of tuberculosis, 2000–2021: an observational study
Source: Front Public Health. 2025 Aug 13;13:1572422. doi: 10.3389/fpubh.2025.1572422 (PMC12380760; doi:10.3389/fpubh.2025.1572422)
Supplement: Supplementary file 1 [file Data_Sheet_1.pdf]

Supplementary Table 1. Diagnostics of quasi-Poisson generalized linear model

Test Item	Value	Note
**Residual Deviance**	41208.2396	
**Residual Degrees of Freedom**	2624	
**Deviance/DF Ratio**	15.7043596	
**Dispersion Parameter**	20.7162702	Dispersion parameter > 1.5, indicating overdispersion, quasi-Poisson model is appropriate
**VIF Value**		
AT	1.11332684	Acceptable
AP	1.11705396	Acceptable
SDI	5.14532049	High multicollinearity
Year	1.06654235	Acceptable
ACT	4.05119974	Acceptable
CHE	1.50182187	Acceptable
POD	1.98342702	Acceptable
PBW	3.66485532	Acceptable
PBS	5.20959645	High multicollinearity
**Adjusted R-squared**	0.75654615	Suggesting that the model explained a high proportion of the variance in tuberculosis outcomes.
