# Supplementary material for: The influence and lag-effect of temperature and precipitation on the incidence and mortality of tuberculosis, 2000–2021: an observational study
Source: Front Public Health. 2025 Aug 13;13:1572422. doi: 10.3389/fpubh.2025.1572422 (PMC12380760; doi:10.3389/fpubh.2025.1572422)
Supplement: Supplementary file 2 [file Data_Sheet_2.pdf]

		Incidence Rate		Mortality Rate	
Sex	Age	EAPC	(95% CI)	EAPC	(95% CI)
African Region					
Both	<5 years	-4.11	(-4.22, -3.99)	-6.24	(-6.56, -5.93)
	5-14 years	-3.15	(-3.35, -2.94)	-4.41	(-4.75, -4.07)
	15-49 years	-1.8	(-1.98, -1.61)	-4.03	(-4.26, -3.8)
	50-69 years	-3.83	(-3.95, -3.7)	-3.97	(-4.09, -3.85)
	70+ years	-3.05	(-3.16, -2.94)	-3.22	(-3.33, -3.11)
Female	<5 years	-4.28	(-4.4, -4.16)	-6.48	(-6.8, -6.16)
	5-14 years	-3.42	(-3.72, -3.13)	-4.26	(-4.64, -3.89)
	15-49 years	-2.57	(-2.84, -2.3)	-4.7	(-5.02, -4.39)
	50-69 years	-4.25	(-4.42, -4.09)	-4.1	(-4.2, -3.99)
	70+ years	-3.15	(-3.29, -3)	-3.07	(-3.22, -2.92)
Male	<5 years	-3.83	(-3.94, -3.72)	-5.93	(-6.25, -5.62)
	5-14 years	-2.75	(-2.86, -2.64)	-4.57	(-4.88, -4.27)
	15-49 years	-0.99	(-1.12, -0.85)	-3.65	(-3.84, -3.45)
	50-69 years	-3.44	(-3.54, -3.34)	-3.77	(-3.9, -3.64)
	70+ years	-2.94	(-3.02, -2.86)	-3.29	(-3.37, -3.2)
Eastern Mediterranean Region					
Both	<5 years	-3.02	(-3.07, -2.97)	-3.33	(-3.6, -3.07)
	5-14 years	-2.98	(-3.3, -2.66)	-3.49	(-4, -2.98)
	15-49 years	-2.69	(-2.77, -2.61)	-3.4	(-3.53, -3.27)
	50-69 years	-3.62	(-3.7, -3.55)	-4.72	(-4.83, -4.6)
	70+ years	-3.51	(-3.6, -3.43)	-4.01	(-4.11, -3.91)
Female	<5 years	-2.96	(-3.01, -2.9)	-3.18	(-3.46, -2.91)
	5-14 years	-2.88	(-3.21, -2.56)	-3.31	(-3.86, -2.76)
	15-49 years	-2.76	(-2.83, -2.69)	-3.54	(-3.67, -3.41)
	50-69 years	-3.41	(-3.48, -3.33)	-4.59	(-4.71, -4.46)
	70+ years	-3.05	(-3.08, -3.02)	-3.65	(-3.73, -3.57)
Male	<5 years	-3.15	(-3.22, -3.08)	-3.57	(-3.83, -3.3)
	5-14 years	-3.15	(-3.47, -2.84)	-3.79	(-4.23, -3.35)
	15-49 years	-2.62	(-2.74, -2.5)	-3.31	(-3.45, -3.17)
	50-69 years	-3.77	(-3.85, -3.7)	-4.8	(-4.91, -4.68)
	70+ years	-3.79	(-3.91, -3.67)	-4.19	(-4.31, -4.06)
European Region					
Both	<5 years	-3.71	(-3.93, -3.48)	-6.97	(-7.31, -6.62)
	5-14 years	-2.5	(-2.8, -2.2)	-7.52	(-7.88, -7.15)
	15-49 years	-4.91	(-5.33, -4.5)	-8.35	(-9.17, -7.54)
	50-69 years	-4.53	(-4.92, -4.13)	-7.1	(-7.67, -6.53)
	70+ years	-4.11	(-4.29, -3.93)	-5.09	(-5.25, -4.94)
Female	<5 years	-3.8	(-4, -3.59)	-6.89	(-7.25, -6.54)
	5-14 years	-2.88	(-3.11, -2.65)	-7.27	(-7.56, -6.98)
	15-49 years	-3.81	(-4.21, -3.41)	-6.81	(-7.88, -5.74)
	50-69 years	-3.71	(-4.02, -3.41)	-5.83	(-6.42, -5.24)
	70+ years	-3.89	(-4.04, -3.74)	-4.3	(-4.45, -4.15)
Male	<5 years	-3.61	(-3.86, -3.35)	-7.03	(-7.39, -6.67)
	5-14 years	-2.07	(-2.46, -1.68)	-7.75	(-8.25, -7.25)
	15-49 years	-5.39	(-5.86, -4.91)	-8.79	(-9.56, -8.03)
	50-69 years	-4.85	(-5.28, -4.43)	-7.41	(-7.99, -6.84)
	70+ years	-4.54	(-4.74, -4.34)	-5.95	(-6.16, -5.75)
Region of the Americas					
Both	<5 years	-3.55	(-3.98, -3.13)	-6.41	(-6.85, -5.98)

	5-14 years	-3.44	(-4.15, -2.73)	-4.94	(-5.37, -4.51)
	15-49 years	-1.18	(-1.41, -0.96)	-3.06	(-3.48, -2.64)
	50-69 years	-2.12	(-2.44, -1.81)	-3.71	(-4.15, -3.27)
	70+ years	-2.55	(-2.62, -2.48)	-3.76	(-3.94, -3.58)
Female	<5 years	-3.63	(-4.12, -3.13)	-6.51	(-6.97, -6.06)
	5-14 years	-3.5	(-4.21, -2.8)	-4.94	(-5.37, -4.5)
	15-49 years	-1.74	(-1.98, -1.5)	-3.85	(-4.26, -3.43)
	50-69 years	-2.31	(-2.64, -1.97)	-4.26	(-4.77, -3.76)
	70+ years	-2.46	(-2.53, -2.39)	-3.77	(-3.95, -3.6)
Male	<5 years	-3.48	(-3.83, -3.12)	-6.33	(-6.76, -5.89)
	5-14 years	-3.36	(-4.07, -2.64)	-4.94	(-5.39, -4.5)
	15-49 years	-0.82	(-1.04, -0.61)	-2.66	(-3.08, -2.25)
	50-69 years	-2.01	(-2.31, -1.7)	-3.46	(-3.87, -3.05)
	70+ years	-2.75	(-2.82, -2.68)	-3.9	(-4.09, -3.71)
South-East Asia Region					
Both	<5 years	-4.3	(-4.48, -4.11)	-7.79	(-8.04, -7.55)
	5-14 years	-4.46	(-4.64, -4.28)	-7.42	(-7.82, -7.02)
	15-49 years	-2.45	(-2.64, -2.25)	-4.86	(-5.06, -4.65)
	50-69 years	-2.58	(-2.7, -2.45)	-4.74	(-4.86, -4.63)
	70+ years	-2.79	(-2.98, -2.6)	-4.36	(-4.58, -4.14)
Female	<5 years	-4.51	(-4.67, -4.34)	-8.11	(-8.37, -7.84)
	5-14 years	-4.83	(-5.05, -4.6)	-7.82	(-8.29, -7.35)
	15-49 years	-2.88	(-3.11, -2.66)	-5.26	(-5.48, -5.04)
	50-69 years	-2.56	(-2.69, -2.42)	-4.7	(-4.95, -4.44)
	70+ years	-2.7	(-2.9, -2.5)	-4.36	(-4.64, -4.09)
Male	<5 years	-4.02	(-4.24, -3.81)	-7.44	(-7.67, -7.21)
	5-14 years	-3.98	(-4.14, -3.81)	-6.9	(-7.4, -6.4)
	15-49 years	-2.04	(-2.21, -1.87)	-4.6	(-4.87, -4.33)
	50-69 years	-2.55	(-2.67, -2.43)	-4.72	(-4.85, -4.59)
	70+ years	-2.79	(-2.97, -2.62)	-4.27	(-4.46, -4.08)
Western Pacific Region					
Both	<5 years	-1.76	(-2.45, -1.07)	-9.18	(-9.4, -8.97)
	5-14 years	-1.63	(-2.19, -1.08)	-6.14	(-6.3, -5.98)
	15-49 years	-1.15	(-1.36, -0.95)	-4.23	(-4.63, -3.83)
	50-69 years	-2.4	(-2.61, -2.18)	-5.63	(-5.99, -5.27)
	70+ years	-2.99	(-3.14, -2.85)	-5.92	(-6.15, -5.69)
Female	<5 years	-1.61	(-2.25, -0.96)	-9.76	(-10, -9.51)
	5-14 years	-2.15	(-2.6, -1.69)	-6.39	(-6.53, -6.25)
	15-49 years	-1.94	(-2.22, -1.66)	-5.18	(-5.73, -4.62)
	50-69 years	-2.51	(-2.78, -2.24)	-6.42	(-6.88, -5.95)
	70+ years	-2.77	(-2.97, -2.58)	-5.89	(-6.14, -5.64)
Male	<5 years	-1.92	(-2.67, -1.18)	-8.69	(-8.9, -8.48)
	5-14 years	-1.04	(-1.71, -0.38)	-5.9	(-6.11, -5.69)
	15-49 years	-0.65	(-0.81, -0.5)	-3.77	(-4.08, -3.46)
	50-69 years	-2.29	(-2.48, -2.1)	-5.25	(-5.56, -4.93)
	70+ years	-3.2	(-3.33, -3.07)	-6.01	(-6.25, -5.77)
