# Supplementary material for: The influence and lag-effect of temperature and precipitation on the incidence and mortality of tuberculosis, 2000–2021: an observational study
Source: Front Public Health. 2025 Aug 13;13:1572422. doi: 10.3389/fpubh.2025.1572422 (PMC12380760; doi:10.3389/fpubh.2025.1572422)
Supplement: Supplementary file 3 [file Data_Sheet_3.docx]

**Supplementary Table 3.** Characteristics of Covariates by different regions, 2000-2021. Values are median (interquartile range) unless stated otherwise.

| **Covariates** | **Total** | **WHO Regions** | | | | | |
| --- | --- | --- | --- | --- | --- | --- | --- |
|  |  | **African Region** | **Region of the Americas** | **Eastern Mediterranean Region** | **European Region** | **South-East Asia Region** | **Western Pacific Region** |
| **AT** | 23.78 (13.72 to 27.28) | 25.55 (22.5 to 27.66) | 23.3 (16.42 to 27.05) | 24.84 (20.38 to 27.2) | 12.87 (9.8 to 19.86) | 26.52 (22.35 to 27.62) | 27.23 (25.65 to 28.08) |
| **AP** | 1.89 (0.82 to 3.12) | 2.05 (0.92 to 3.01) | 2.02 (1.25 to 3.12) | 0.5 (0.14 to 2.18) | 1.43 (0.69 to 2.23) | 2.98 (1.16 to 4.08) | 3.15 (2.33 to 5.13) |
| **SDI** | 0.61 (0.45 to 0.74) | 0.38 (0.29 to 0.48) | 0.62 (0.56 to 0.68) | 0.61 (0.48 to 0.71) | 0.78 (0.71 to 0.83) | 0.48 (0.4 to 0.6) | 0.62 (0.54 to 0.79) |
| **ACT** | 85.3 (26.4 to 100) | 7.6 (1.4 to 36) | 94 (83.3 to 100) | 98.9 (90.1 to 100) | 100 (94.9 to 100) | 37.2 (16.8 to 74) | 72.5 (25.35 to 100) |
| **CHE** | 127.12 (22.21 to 595.72) | 10.99 (5.75 to 41.63) | 221.49 (109.88 to 452.46) | 83.81 (28.83 to 413.24) | 882.5 (209 to 3042.49) | 21 (6 to 76) | 197.24 (74.89 to 647.82) |
| **POD** | 1.2 (0 to 11.51) | 18.32 (7.37 to 35.93) | 2.15 (0.8 to 6.23) | 0.47 (0.01 to 11.19) | 0 (0 to 0.03) | 11.94 (1.3 to 29.82) | 0.37 (0 to 5.01) |
| **PBW** | 94.19 (77.53 to 99.23) | 59.44 (48.06 to 73.6) | 94.66 (91.17 to 98.37) | 96.86 (88.05 to 98.94) | 99.86 (96.79 to 100) | 93.35 (84.91 to 97.16) | 97.65 (90.38 to 99.85) |
| **PBS** | 86.35 (48.4 to 97.67) | 30.42 (16.6 to 48.01) | 85.63 (77.07 to 93.38) | 91.75 (71.94 to 98.59) | 98.05 (95.31 to 99.4) | 62.04 (42.27 to 84.07) | 91.55 (76.08 to 99.91) |

AT, National annual average temperature (°C); AP, National annual average precipitation (mm); ACT, Access to clean fuels and technologies for cooking (% of population); CHE, Domestic general government health expenditure per capita (current US$); POD, People practicing open defecation (% of population); PBW, People using at least basic drinking water services (% of population); PBS, People using at least basic sanitation services (% of population).
